# Gastrointestinal tract anastomoses with the biofragmentable anastomosis ring: is it still a valid technique for bowel anastomosis? Analysis of 203 cases and review of the literature

**DOI:** 10.1007/s00384-016-2661-z

**Published:** 2016-09-30

**Authors:** Adam Bobkiewicz, Adam Studniarek, Lukasz Krokowicz, Krzysztof Szmyt, Maciej Borejsza-Wysocki, Jacek Szmeja, Ryszard Marciniak, Michal Drews, Tomasz Banasiewicz

**Affiliations:** Department of General, Endocrinological Surgery and Gastroenterological Oncology, Poznan University of Medical Sciences, Przybyszewskiego 49, 60-355 Poznan, Poland

**Keywords:** Biofragmentable anastomosis ring, Sutureless anastomosis, Compression anastomosis, Bowel anastomosis

## Abstract

**Purpose:**

Biofragmentable anastomosis ring (BAR) is an alternative to manual and stapled anastomoses performed within the upper and lower gastrointestinal (GI) tract. The aim of this study was to evaluate the effectiveness of BAR utility for bowel anastomoses based on our own material.

**Methods:**

A retrospective analysis was performed to a total of 203 patients who underwent bowel surgery with the use of BAR anastomosis within upper and lower gastrointestinal tract between 2004 and 2014. Data for the analysis was collected based on medical records, treatment protocols, and the results of histological examinations.

**Results:**

The study group consisted of 86 women and 117 men. The most common underlying pathology was a malignant disease (*n* = 165). Biofragmentable anastomosis ring (BAR) size 31 was the most commonly used (*n* = 87). A total of 169 colocolic or colorectal anastomoses and 28 ileocolic and 8 enteroenteric anastomoses were performed. The mortality rate was 0.5 % (*n* = 1) whereas re-surgery rate within 30 days was 8.4 % (*n* = 17). Twenty-eight patients developed perioperative complications with surgical site infection as the most common one (*n* = 11). Eight patients developed specific complications associated with BAR including an anastomotic leak (*n* = 6) and intestinal obstruction (*n* = 2). The mean time of hospital stay after surgery was 12.7 days.

**Conclusions:**

The use of BAR for the GI tract anastomoses is simple and rapid method and it is characterized with an acceptable number of perioperative mortality and complication rates. Based on our experience, we recommend the use of BAR anastomosis in different types of intestinal anastomosis in varying clinical scenarios.

## Introduction

Biofragmentable anastomosis ring (BAR) is a well-known surgical device used for the purpose of bowel anastomosis. It consists of two identical rings composed of absorbable polyglycolic acid (87.5 %) and barium sulfate (12.5 %) acting as radiopaque dyes [1].

Although a number of experimental studies had been performed even earlier, it was in 1892 when Murphy described specially designed device for the compression anastomosis called Murphy’s button [2]. Despite the initial enthusiasm for this procedure, the long-term outcomes were not satisfactory due to high rate of anastomotic stenosis as the most common complication [3].

The introduction of BAR by Hardy et al. initialized the era of compression anastomoses [4]. Although in USA BAR has not been commonly used, in many European countries it is still a method of choice in a number of clinical settings where bowel anastomosis is performed [5]. Currently, there is a wide spectrum of indications for BAR anastomosis. In both upper and lower gastrointestinal (GI) tract, varying types of anastomoses including end-to-end, end-to-side, and side-to-side BAR have been used as a method of choice [6–9].

The main goal of the study was to evaluate the results using BAR anastomoses in 203 patients qualified for colonic or enteric surgery at a single tertiary reference center.

## Methods and materials

Retrospectively, we analyzed a group of 203 patients who underwent a small or large bowel surgery with the use of BAR anastomosis in our institution between 2004 and 2014. Data was collected based on the available medical records. Medical records were retrospectively reviewed in order to analyze patients’ demographics, the indications for surgery and underlying pathology, distribution of BAR sizes, the site of BAR anastomosis as well as intra- and perioperative complications following surgery.

Varying sizes of BARs were used (28, 31, 34). We did not routinely use a BAR size 25. In the clinical situation with the discrepancy of both bowel ends, we preferably used a smaller BAR size compatible with the smaller lumen instead of luminal dilatation of the smaller bowel end. Originally, BAR devices possess three different gap junctions (1.5, 2, and 2.5 mm). In most cases, a BAR with 2-mm gap junction was used whereas a BAR with wider gap junction was used in cases with a thick bowel wall.

## Surgical procedure

Routinely, the day before the surgery both the small bowel and colon were prepared with polyethylene glycol electrolyte lavage solution. Arbitrary patients were counseled preoperatively by stoma nursing team for the purpose of marking the optimal site for a potential stoma creation if needed. Obligatory patients were administered cefazoline and metronidazole as antibiotic prophylaxis which was prolonged over the next 24 h. Preferably, surgery was performed with the midline incision. Since the appropriate dissection was made, purse-string sutures (monofilament absorbable) were placed at the marked bowel ends and the affected part of the bowel was resected. BAR (Valtrac™ BAR, Norwalk, CT, USA) was used as a standard sutureless intestinal anastomosis. All particular steps of the procedure followed a standard technique described by Hardy et al. [4]. The exact BAR diameter was established based on the specially designed sizing instrument. One ring of the BAR was placed into the cut bowel end and secured with purse-string suture. The inserter of BAR was removed and the second ring of the BAR was introduced into another cut bowel end and secured with another purse-string suture. Following a thorough inspection to confirm that there was no tension, serosal apposition and that the mesentery is properly seated, both rings were then snapped to complete the anastomosis. Routinely, patients were left on a full liquid diet since the complete fragmentation of BAR was confirmed based on the plain X-ray of the abdomen. Usually X-ray was done 3 weeks following the surgery and repeated if needed in case of BAR presence within the intestinal lumen.

All described data is presented as mean and standard deviation (mean ± SD). These findings were analyzed using Statistica 10.0 StatSoft software (StatSoft, Inc. Tulsa, USA).

## Results

A total of 204 patients were enrolled into the study with 205 bowel anastomoses constructed using BAR. In one patient with sigmoid carcinoma and invasion of one of the small bowel loops simultaneously two BAR anastomoses were performed (BAR 34 and 28, respectively). The study group consisted of 86 women (42.4 %) and 117 men (57.6 %). The mean age of patients at the time of surgery was 63.5 (SD = 12.4, range from 20 to 88 years). The most common underlying etiology was a malignant disease within the large bowel (*n* = 165). The underlying pathologies are detailed in Table 1. Only three sizes of BAR (28, 31, 34) were routinely used based on our experience and BAR size 31 was used as the most common one (42.6 %). BAR specifications are summarized in Table 2. End-to-end type of anastomoses were performed preferably in all surgical procedures (*n* = 204) because of surgeons’ preferences as well as anatomic considerations. Colocolonic or colorectal anastomoses were the most frequently performed with a total of 169 BARs used followed by ileocolic (*n* = 28) and enteroenteric (*n* = 7) anastomoses. The types of surgical procedures and types of anastomoses are shown in Table 3. Additionally, 26 patients underwent another simultaneous surgery. The most common one was metastatic liver resection (*n* = 6), cholecystectomy (*n* = 6), appendectomy (*n* = 3), abdominal hernia repair (*n* = 3), and others (*n* = 8). The mean time of the surgery was 158.2 ± 55.4 min (ranged from 60 to 435 min). The mean time of postoperative hospital stay was 12.7 days (SD = 11.1, range from 5 to 92 days). There was one perioperative death (0.5 %) due to a dehiscence of anastomotic site, secondary peritonitis, and multiorgan failure. Re-surgery rate within 30 days was 8.3 % (17 patients). Twenty-eight patients developed surgical postoperative complications (Table 4). Eleven of them presented with surgical site infection (SSI) limited to superficial surgical site infection. In majority of patients with SSI, they were treated conservatively. Additionally in four patients, negative pressure wound therapy (NPWT) was implemented. Eight patients developed specific complications associated with BAR anastomosis including anastomotic leak (*n* = 6) and intestinal obstruction (*n* = 2). The mean postoperative day when anastomotic leakage was revealed was 9.8 (SD 3.1). Three patients with intra-abdominal abscesses required re-operation and drainage of the abscesses.

**Table 1:** Underlying pathology in patients with BAR anastomosis

Pathology	No. of patients (*n* = 203)	Percentage (%)
Malignant disease	*n* = 165	81.3
Diverticular disease	*n* = 17	8.4
Multiple colorectal polyps	*n* = 10	4.9
Crohn’s disease	*n* = 2	1.0
Enterocutaneous fistula	*n* = 2	1.0
Others^a^	*n* = 7	3.4

^a^Including gastrointestinal stromal tumor, migration of biomaterial prosthesis, obstipatio chronica, small bowel intussusception, small bowel incarceration, acute gastrointestinal hemorrhage, and leiomyosarcoma of the small and large bowel

**Table 2:** BAR specifications and distributions within bowel surgery

Type of anastomosis	BAR size (mm)				
	28	31	34	NA	Total
Enteroenteric	4	2	1	0	7
Colocolic/colorectal	58	74	26	11	169
Enterocolic	13	11	2	2	28
Total	75	87	29	13	204

*NA* not available

**Table 3:** Type of surgical procedures and number of BAR anastomosis

Type of anastomosis	No. of BAR anastomosis (*n* = 204)	Percentage (%)
Enteroenteric		
Segmental resection	*n* = 7	3.4
Colocolic/colorectal		
Sigmoid resection	*n* = 100	49.0
Left colectomy	*n* = 58	28.4
Segmental resection	*n* = 11	5.4
Ileocolic		
Ileoceacal resection	*n* = 10	4.9
Right hemicolecotmy	*n* = 8	3.9
Total colectomy	*n* = 10	4.9

**Table 4:** Surgical postoperative complications

Complication	No. of patients (*n* = 203)	Percentage (%)
Surgical site infection	*n* = 11	5.4
Wound dehiscence	*n* = 4	2.0
Intra-abdominal abscess	*n* = 3	1.5
Gastrointestinal bleeding	*n* = 2	1.0
Anastomotic leakage	*n* = 6	3.0
Intestinal obstruction	*n* = 2	1.0
Re-surgery rate	17/203	8.4
Perioperative mortality	*n* = 1	0.5

## Discussion

Three crucial principles should be met in every intestinal anastomosis: adequate blood supply, absence of serosal apposition as well as an absence of tension at the anastomotic site [10]. Unquestionably, the biofragmentable nature of the BAR anastomosis structure as well as the material (polyglycolic acid) presents the sufficient profile for intestinal anastomosis [5]. Although the first reports indicated some technical difficulties associated with BAR application, the introduction of specially designed tools such as dilatation device, pure-string clamps, or anastomotic forceps significantly facilitates operative handling of a BAR [11].

Comparative data of the latest studies using BAR regarding postoperative complications were summarized in Table 5. Based on our experience, only eight postoperative complications were present and they were strictly associated with BAR anastomosis (anastomotic leakage, *n* = 6; intestinal obstruction, *n* = 2). One of the most challenging complications following bowel anastomosis associated with high morbidity and mortality is an anastomotic leak. Based on the previous large series, the anastomotic leak rate ranged between 2.5 and 4.2 % [6, 14–16]. Although based on the multivariate analysis the use of BAR was considered to be a risk factor for anastomotic leak after bowel resection, the overall leak rate according to some randomized studies regarding BAR, stapled and hand-sewn anastomosis confirmed comparable leak rates [8, 13, 15]. Based on prospective randomized study, the efficiency, complication rate, and postoperative recovery were comparable in groups using BAR and manual sutures [17]. Based on our study, leak rate occurred in 3.0 % of patients (*n* = 6) which is consistent with other studies mentioned above. All leakages of BAR anastomoses required re-surgery. Hartmann procedure was performed in the majority of cases.

**Table 5:** Results of postoperative complications from large studies using BAR

Study	Year	No. of patients	No. of BAR	Anastomotic leakage (%)	Obstruction (%)	Surgical site infection (%)
Cahill et al. [12]	1989	101	101	2	4	10
Corman et al. [8]	1989	222	222	2.7	4.1	5.0
Bubrick et al. [13]	1991	370	370	3	4.6	5.0
Di Castro et al. [14]	1998	453	514	3.8	0	NA
Thiede et al. [6]	1998	1360	1666	2.5	NA	NA
Choi et al. [15]	1998	140	144	2.9	3.6	6.4
Ghitulescu et al. [16]	2003	165	173	4.2	7.9	0.6
Kim et al. [7]	2005	617	632	0.8	2.1	4.7
Present study	2015	204	205	3.0	1.0	5.4

*NA* not available

Surprisingly, all anastomotic leaks occurred in colocolonic anastomosis (*n* = 6). Similar results were proven by Mokros who reviewed over 1000 BAR anastomoses and found predilection of anastomosis insufficiency within the lower GI tract (4.2 versus 0.4 % in the upper GI tract) [18]. One of the possible explanations is the inadequate approximation of bowel ends because of two large compression zones of the large bowel wall. It was also suggested by Theide et al. that bowel wall thickness might be the risk factor for technical problems with BAR compression resulting in further anastomotic dehiscence [6].

Cossu et al. presented a study reporting the utility of multiple (single, double, and triple) BARs for bowel anastomoses within both the upper and lower GI tract [19]. We performed multiple BAR anastomoses only in one patient; thus, the real value of such management is difficult to assess. However, the majority of BAR anastomoses were performed within upper part of the GI tract which predisposes for faster resumption of transit and intestinal canalization [18, 19].

The overall incidence rate of surgical site infection following bowel surgery was observed to range between 5.8 and 17.9 % [20–22]. SSI rate following bowel surgery using BAR anastomosis was reported to be lower and ranging between 4.7 and 5.0 % in large series [7, 8, 13]. These results are comparable with our experiences (5.4 %). In four patients, NPWT was used as a support for SSI management resulting in a faster healing rate. It was a widely accepted technique which significantly increased the efficiency of treatment of SSI [23, 24].

Our observations regarding some technical aspects during BAR application are comparable to these presented by Forde et al. [5]. It is necessary to apply BAR with a larger gap junction in cases with edematous and thick tissues. We also do not recommend the use of BAR anastomosis in inflamed bowel such a Crohn’s disease or ulcerative colitis. Usually, the general condition and metabolic status in these patients are poor; therefore, the healing process within anastomotic ring may be impaired and prolonged which may result in an incomplete anastomotic site healing since the defragmentation occurs. Due to technical difficulties, we did not routinely perform BAR anastomosis within the rectum. Although Chen et al. presented some modifications of BAR with the intention to utilize it for rectal management, this method still possessed some limitations and was not introduced to routine clinical practice [25, 26]. However, Galizia et al. proved the feasibility and safety of BAR for the extraperitoneal rectal surgery [27]. Moreover, the outcomes were comparable to the stapled technique. Structure and natural history of BAR anastomosis predispose for lower rate of intestinal stenosis. According to the results of large studies (more than 100 patients) concerning BAR anastomosis, late stricture rate did not exceed 2 % [7, 8, 16]. It is believed to be associated with a shorter inflammation phase in response to a foreign body within the anastomotic site and a deposition of collagen tissue [11, 28]. Based on our experience, we did not observe any late intestinal strictures. However, in early postoperative course (within 30 days), bowel obstruction was observed in two patients which was associated with overly rapid implementation of solid food diet. Conservative treatment in both cases brought relief and resolution of symptoms.

This study has several limitations. First, generalizability of the study conclusion must be approached with caution because of the single-center study and the retrospective nature of the study design. Medical data were collected at the time of routine clinical care and are based on real-life observations. As such, patients were not randomized and no matched controls were performed. Moreover, there is no homogenous population regarding underlying pathology as well as type of surgical procedures. Second, there is missing data of follow-up. Thus, the rate of long-term procedure-related complications are unknown, especially those associated with late anastomotic stricture that was reported by others. Third, we had no precise medical records regarding the time required to construct BAR anastomosis. It may interestingly show the real rapidity of the anastomotic construction that was indicated in previous studies. Fourth, we used BAR anastomosis only in elective surgery.

However, we present data on a relatively large population of patients that are largely in the line with the results of previous studies.

## Conclusions

Safety and efficiency of a BAR anastomosis depend on appropriate preparation and positioning of the anastomosed intestinal ends. Adequate blood supply, absence of tension, and serosal apposition are the main criteria to create a proper, well-functioning anastomosis. Based on our experience, we recommend the use of BAR anastomosis in different types of intestinal anastomosis in varying clinical scenarios. Technical simplicity of versatility and rapidity makes a BAR technique still an attractive alternative to other types of bowel anastomoses.
